# Supplementary material for: Design, Synthesis, and Structure–Activity Relationship Studies of New Quinone Derivatives as Antibacterial Agents
Source: Antibiotics (Basel). 2023 Jun 16;12(6):1065. doi: 10.3390/antibiotics12061065 (PMC10295782; doi:10.3390/antibiotics12061065)

antibiotics

Supplementary materials

# Design, synthesis, and structure-activity relationship studies of new quinone derivatives as antibacterial agents

Juan Andrades-Lagos [1,2†*], Javier Campanini-Salinas [2,3†*], América Pedreros-Riquelme [2], Jaime Mella [4,5], Duane Choquesillo-Lazarte [6], P.P. Zamora [7], Hernán Pessoa-Mahana [8]; Ian Burbulis [9] and David Vásquez-Velásquez [2,*]

1 Table

11 Figures

26 Pages

## GENERAL SYNTHESIS PROCEDURES PRESENT IN FIGURE 3, INDIVIDUALIZED.

**FIGURE S1**

General Procedure B

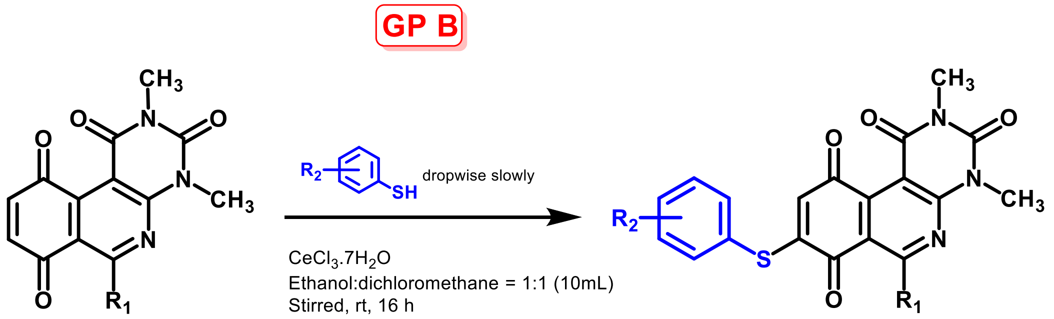


**5: $R_1$ = Et**
**6: $R_1$ = Me**
**7: $R_1$ = H**

**$R_1$ = Et: 7-9, 11-21, 38-41**
**$R_1$ = Me: 42-44**

**FIGURE S2**

General Procedure B

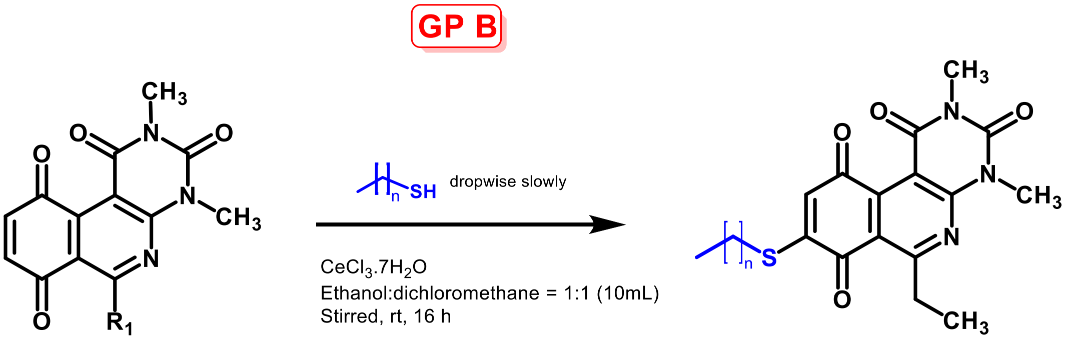


**5: $R_1$ = Et**

**30-34**

**FIGURE S3**

General Procedure C

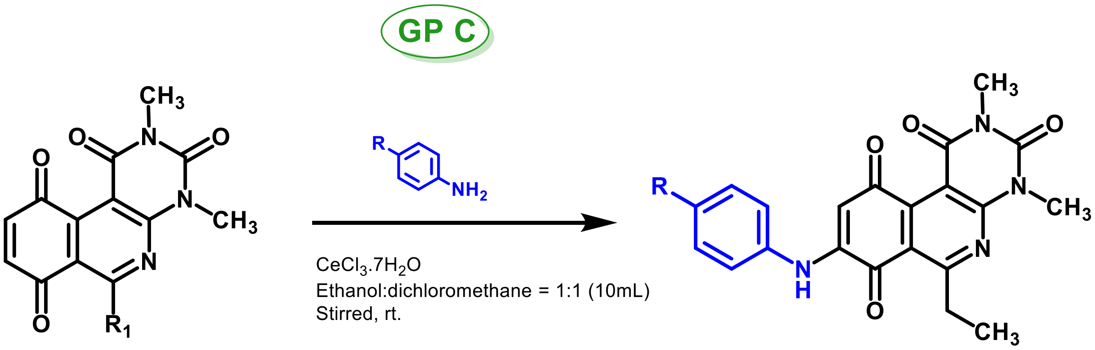


**5: $R_1$ = Et**

**22, 24, 26-29**

**FIGURE S4**

General Procedure D

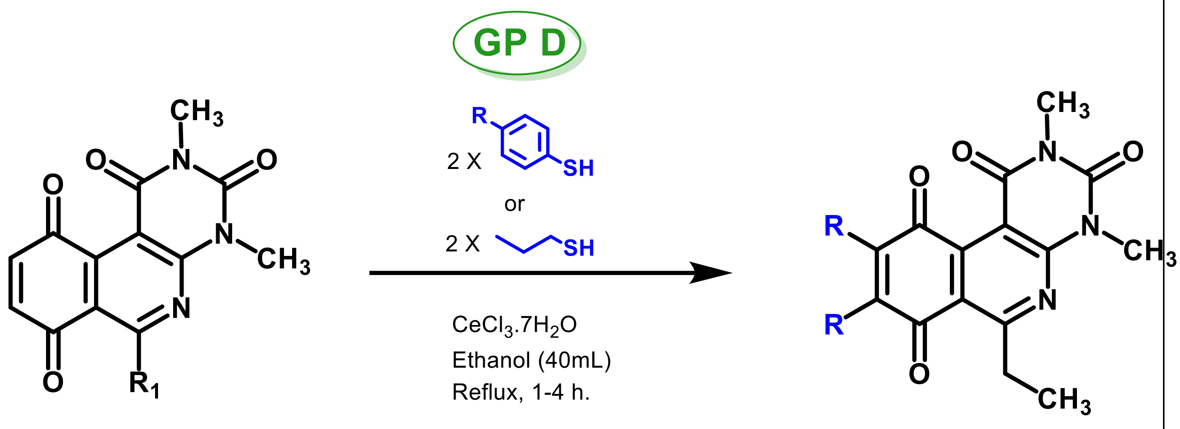


5: $R_1$ = Et

35-37

**TABLE S1. CRYSTALLOGRAPHIC INFORMATION**

| | **5c.cif** | **6c.cif** | **7c.cif** | **8c.cif** | **11c.cif** | **19c.cif** | **20c.cif** |
|---|---|---|---|---|---|---|---|
| Figure | S5 | S6 | S7 | S8 | S9 | S10 | S11 |
| Empirical formula | $C_{21}H_{15}BrClN_3O_4S$ | $C_{17}H_{17}N_3O_4S$ | $C_{21}H_{17}ClN_4O_4$ | $C_{21}H_{17}BrN_4O_4$ | $C_{20}H_{14}BrN_3O_4S$ | $C_{19}H_{21}N_3O_4S$ | $C_{21}H_{19}N_5O_4$ |
| Formula weight | 520.78 | 359.39 | 424.84 | 469.30 | 472.31 | 387.45 | 405.41 |
| Temperature [K] | 298(2) | 298(2) | 298(2) | 298(2) | 298(2) | 298(2) | 298(2) |
| Crystal system | monoclinic | triclinic | monoclinic | monoclinic | orthorhombic | monoclinic | monoclinic |
| Space group (number) | $P2_1/n$ (14) | $P\overline{1}$ (2) | $P2_1/c$ (14) | $P2_1/c$ (14) | $Pbcn$ (60) | $P2_1/n$ (14) | $P2_1/c$ (14) |
| *a* [Å] | 9.0790(11) | 4.4062(8) | 7.6795(2) | 7.6848(2) | 24.2195(6) | 4.5196(2) | 8.7845(3) |
| *b* [Å] | 7.8903(17) | 10.9278(19) | 16.6845(4) | 16.8295(5) | 7.5475(2) | 26.8428(13) | 10.4767(4) |
| *c* [Å] | 28.412(3) | 17.524(3) | 15.1553(5) | 15.2751(5) | 21.3877(6) | 15.3585(10) | 21.3483(10) |
| α [°] | 90 | 80.857(5) | 90 | 90 | 90 | 90 | 90 |
| β [°] | 90.884(4) | 88.521(6) | 95.638(2) | 95.860(2) | 90 | 95.006(4) | 99.351(3) |
| γ [°] | 90 | 83.122(6) | 90 | 90 | 90 | 90 | 90 |
| Volume [Å$^3$] | 2035.1(5) | 827.1(3) | 1932.43(9) | 1965.23(10) | 3909.60(18) | 1856.17(17) | 1938.63(14) |
| *Z* | 4 | 2 | 4 | 4 | 8 | 4 | 4 |
| $\rho_{calc}$ [gcm$^{-3}$] | 1.700 | 1.443 | 1.460 | 1.586 | 1.605 | 1.386 | 1.389 |
| $\mu$ [mm$^{-1}$] | 2.290 | 0.224 | 2.080 | 3.177 | 4.156 | 1.816 | 0.821 |
| *F*(000) | 1048 | 376 | 880 | 952 | 1904 | 816 | 848 |
| Reflections collected | 16317 | 11000 | 22651 | 38361 | 19486 | 18750 | 36846 |
| Independent reflections | 4678 | 2899 | 3405 | 3443 | 3427 | 3269 | 3426 |
| Completeness | 99.5 % | 99.1 % | 99.6 % | 99.4 % | 99.2 % | 98.5 % | 99.2 % |
| Data / Restraints / Parameters | 4678/0/283 | 2899/0/230 | 3405/0/275 | 3443/0/275 | 3427/0/266 | 3269/0/249 | 3426/0/276 |
| Goodness-of-fit on $F^2$ | 1.034 | 1.031 | 1.062 | 1.074 | 1.090 | 1.046 | 1.043 |
| Final *R* indexes [$I \geq 2\sigma(I)$] | $R_1$ = 0.0323 w$R_2$ = 0.0824 | $R_1$ = 0.0836 w$R_2$ = 0.1503 | $R_1$ = 0.0684 w$R_2$ = 0.1497 | $R_1$ = 0.0396 w$R_2$ = 0.1001 | $R_1$ = 0.0654 w$R_2$ = 0.1397 | $R_1$ = 0.0441 w$R_2$ = 0.1083 | $R_1$ = 0.0481 w$R_2$ = 0.1310 |
| Final *R* indexes [all data] | $R_1$ = 0.0466 w$R_2$ = 0.0888 | $R_1$ = 0.2047 w$R_2$ = 0.1864 | $R_1$ = 0.1194 w$R_2$ = 0.1789 | $R_1$ = 0.0469 w$R_2$ = 0.1059 | $R_1$ = 0.1055 w$R_2$ = 0.1621 | $R_1$ = 0.0557 w$R_2$ = 0.1155 | $R_1$ = 0.0508 w$R_2$ = 0.1334 |
| Largest peak/hole [eÅ$^{-3}$] | 0.42/-0.59 | 0.52/-0.21 | 0.29/-0.27 | 0.46/-0.65 | 0.44/-0.64 | 0.21/-0.20 | 0.29/-0.23 |

Figure S5

# checkCIF/PLATON report

You have not supplied any structure factors. As a result the full set of tests cannot be run.

THIS REPORT IS FOR GUIDANCE ONLY. IF USED AS PART OF A REVIEW PROCEDURE FOR PUBLICATION, IT SHOULD NOT REPLACE THE EXPERTISE OF AN EXPERIENCED CRYSTALLOGRAPHIC REFEREE.

No syntax errors found. CIF dictionary Interpreting this report

# Datablock: 5c

```
Bond precision:  C-C = 0.0031 A          Wavelength=0.71073

Cell:            a=9.0790(11)     b=7.8903(17)       c=28.412(3)
                 alpha=90         beta=90.884(4)     gamma=90
Temperature:     298 K
```

| | Calculated | Reported |
|---|---|---|
| Volume | 2035.1(5) | 2035.1(5) |
| Space group | P 21/n | P2(1)/n |
| Hall group | -P 2yn | -P 2yn |
| Moiety formula | C21 H15 Br Cl N3 O4 S | C21 H15 Br Cl N3 O4 S |
| Sum formula | C21 H15 Br Cl N3 O4 S | C21 H15 Br Cl N3 O4 S |
| Mr | 520.77 | 520.78 |
| Dx,g cm-3 | 1.700 | 1.700 |
| Z | 4 | 4 |
| Mu (mm-1) | 2.290 | 2.290 |
| F000 | 1048.0 | 1048.0 |
| F000’ | 1048.34 | |
| h,k,lmax | 11,10,37 | 11,10,36 |
| Nref | 4721 | 4678 |
| Tmin,Tmax | 0.760,0.795 | 0.508,0.746 |
| Tmin’ | 0.760 | |

```
Correction method= # Reported T Limits: Tmin=0.508 Tmax=0.746
AbsCorr = MULTI-SCAN

Data completeness= 0.991          Theta(max)= 27.591

R(reflections)= 0.0323( 3751)     wR2(reflections)= 0.0888( 4678)

S = 1.034                   Npar= 283
```

The following ALERTS were generated. Each ALERT has the format
**test-name_ALERT_alert-type_alert-level**.
Click on the hyperlinks for more details of the test.

Figure S5

**Alert level C**

```
PLAT431_ALERT_2_C Short Inter HL..A Contact  Br12     ..O15      .       3.12 Ang.
                                                  x,1+y,z  =      1_565 Check
```

**Alert level G**

```
PLAT883_ALERT_1_G No Info/Value for _atom_sites_solution_primary .     Please Do !
PLAT941_ALERT_3_G Average HKL Measurement Multiplicity ..........        3.5 Low
```

```
   0 ALERT level A = Most likely a serious problem - resolve or explain
   0 ALERT level B = A potentially serious problem, consider carefully
   1 ALERT level C = Check. Ensure it is not caused by an omission or oversight
   2 ALERT level G = General information/check it is not something unexpected

   1 ALERT type 1 CIF construction/syntax error, inconsistent or missing data
   1 ALERT type 2 Indicator that the structure model may be wrong or deficient
   1 ALERT type 3 Indicator that the structure quality may be low
   0 ALERT type 4 Improvement, methodology, query or suggestion
   0 ALERT type 5 Informative message, check
```

It is advisable to attempt to resolve as many as possible of the alerts in all categories. Often the minor alerts point to easily fixed oversights, errors and omissions in your CIF or refinement strategy, so attention to these fine details can be worthwhile. In order to resolve some of the more serious problems it may be necessary to carry out additional measurements or structure refinements. However, the purpose of your study may justify the reported deviations and the more serious of these should normally be commented upon in the discussion or experimental section of a paper or in the "special_details" fields of the CIF. checkCIF was carefully designed to identify outliers and unusual parameters, but every test has its limitations and alerts that are not important in a particular case may appear. Conversely, the absence of alerts does not guarantee there are no aspects of the results needing attention. It is up to the individual to critically assess their own results and, if necessary, seek expert advice.

**Publication of your CIF in IUCr journals**

A basic structural check has been run on your CIF. These basic checks will be run on all CIFs submitted for publication in IUCr journals (*Acta Crystallographica*, *Journal of Applied Crystallography*, *Journal of Synchrotron Radiation*); however, if you intend to submit to *Acta Crystallographica Section C* or *E* or *IUCrData*, you should make sure that full publication checks are run on the final version of your CIF prior to submission.

**Publication of your CIF in other journals**

Please refer to the *Notes for Authors* of the relevant journal for any special instructions relating to CIF submission.

**PLATON version of 10/08/2020; check.def file version of 06/08/2020**

Figure S5

**Datablock 5c** - ellipsoid plot

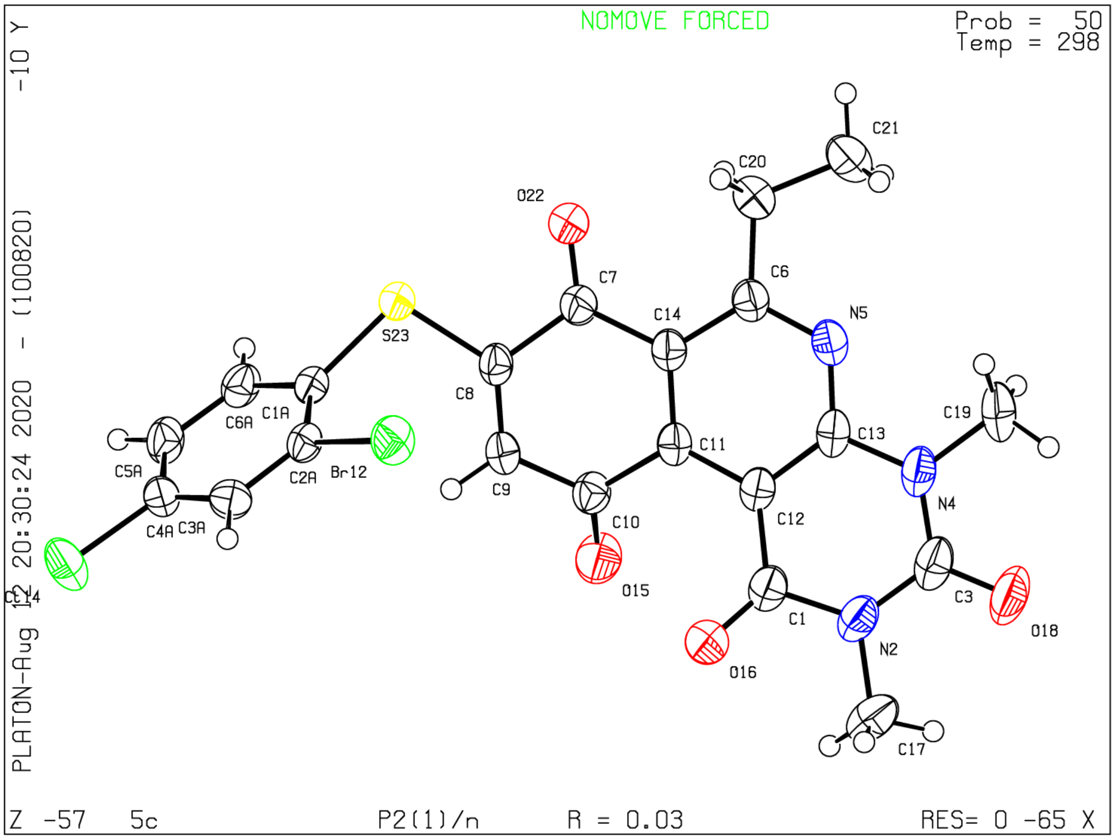

Figure S6 **checkCIF/PLATON report**

You have not supplied any structure factors. As a result the full set of tests cannot be run.

THIS REPORT IS FOR GUIDANCE ONLY. IF USED AS PART OF A REVIEW PROCEDURE FOR PUBLICATION, IT SHOULD NOT REPLACE THE EXPERTISE OF AN EXPERIENCED CRYSTALLOGRAPHIC REFEREE.

No syntax errors found. CIF dictionary Interpreting this report

## Datablock: 6c

---

```
Bond precision:  C-C = 0.0073 A        Wavelength=0.71073

Cell:          a=4.4062(8)       b=10.9278(19)    c=17.524(3)
               alpha=80.857(5)   beta=88.521(6)   gamma=83.122(6)
Temperature:   298 K

                Calculated                Reported
Volume          827.1(3)                  827.1(3)
Space group     P -1                      P -1
Hall group      -P 1                      -P 1
Moiety formula  C17 H17 N3 O4 S           C17 H17 N3 O4 S
Sum formula     C17 H17 N3 O4 S           C17 H17 N3 O4 S
Mr              359.40                    359.39
Dx,g cm-3       1.443                     1.443
Z               2                         2
Mu (mm-1)       0.224                     0.224
F000            376.0                     376.0
F000'           376.42
h,k,lmax        5,12,20                   5,12,20
Nref            2923                      2899
Tmin,Tmax       0.971,0.976               0.484,0.745
Tmin'           0.971

Correction method= # Reported T Limits: Tmin=0.484 Tmax=0.745
AbsCorr = MULTI-SCAN

Data completeness= 0.992          Theta(max)= 25.008

R(reflections)= 0.0836( 1308)     wR2(reflections)= 0.1864( 2899)

S = 1.031                 Npar= 230
```

---

The following ALERTS were generated. Each ALERT has the format
**test-name_ALERT_alert-type_alert-level**.
Click on the hyperlinks for more details of the test.

Figure S6

```
Alert level B
RINTA01_ALERT_3_B  The value of Rint is greater than 0.18
            Rint given   0.184
PLAT020_ALERT_3_B The Value of Rint is Greater Than 0.12 .........      0.184 Report
```

```
Alert level C
PLAT026_ALERT_3_C Ratio Observed / Unique Reflections (too) Low ..        45% Check
PLAT094_ALERT_2_C Ratio of Maximum / Minimum Residual Density ....       2.54 Report
PLAT340_ALERT_3_C Low Bond Precision on  C-C Bonds ...............    0.00731 Ang.
```

```
Alert level G
PLAT883_ALERT_1_G No Info/Value for _atom_sites_solution_primary .     Please Do !
PLAT941_ALERT_3_G Average HKL Measurement Multiplicity ...........        3.8 Low
```

```
   0 ALERT level A = Most likely a serious problem - resolve or explain
   2 ALERT level B = A potentially serious problem, consider carefully
   3 ALERT level C = Check. Ensure it is not caused by an omission or oversight
   2 ALERT level G = General information/check it is not something unexpected

   1 ALERT type 1 CIF construction/syntax error, inconsistent or missing data
   1 ALERT type 2 Indicator that the structure model may be wrong or deficient
   5 ALERT type 3 Indicator that the structure quality may be low
   0 ALERT type 4 Improvement, methodology, query or suggestion
   0 ALERT type 5 Informative message, check
```

### Validation response form

Please find below a validation response form (VRF) that can be filled in and pasted into your CIF.

```
# start Validation Reply Form
_vrf_RINTA01_6c
;
PROBLEM: The value of Rint is greater than 0.18
RESPONSE: ...
;
_vrf_PLAT020_6c
;
PROBLEM: The Value of Rint is Greater Than 0.12 .........      0.184 Report
RESPONSE: ...
;
# end Validation Reply Form
```

Figure S6

It is advisable to attempt to resolve as many as possible of the alerts in all categories. Often the minor alerts point to easily fixed oversights, errors and omissions in your CIF or refinement strategy, so attention to these fine details can be worthwhile. In order to resolve some of the more serious problems it may be necessary to carry out additional measurements or structure refinements. However, the purpose of your study may justify the reported deviations and the more serious of these should normally be commented upon in the discussion or experimental section of a paper or in the "special_details" fields of the CIF. checkCIF was carefully designed to identify outliers and unusual parameters, but every test has its limitations and alerts that are not important in a particular case may appear. Conversely, the absence of alerts does not guarantee there are no aspects of the results needing attention. It is up to the individual to critically assess their own results and, if necessary, seek expert advice.

**Publication of your CIF in IUCr journals**

A basic structural check has been run on your CIF. These basic checks will be run on all CIFs submitted for publication in IUCr journals (*Acta Crystallographica*, *Journal of Applied Crystallography*, *Journal of Synchrotron Radiation*); however, if you intend to submit to *Acta Crystallographica Section C* or *E* or *IUCrData*, you should make sure that full publication checks are run on the final version of your CIF prior to submission.

**Publication of your CIF in other journals**

Please refer to the *Notes for Authors* of the relevant journal for any special instructions relating to CIF submission.

---

**PLATON version of 10/08/2020; check.def file version of 06/08/2020**

Figure S6

**Datablock 6c** - ellipsoid plot

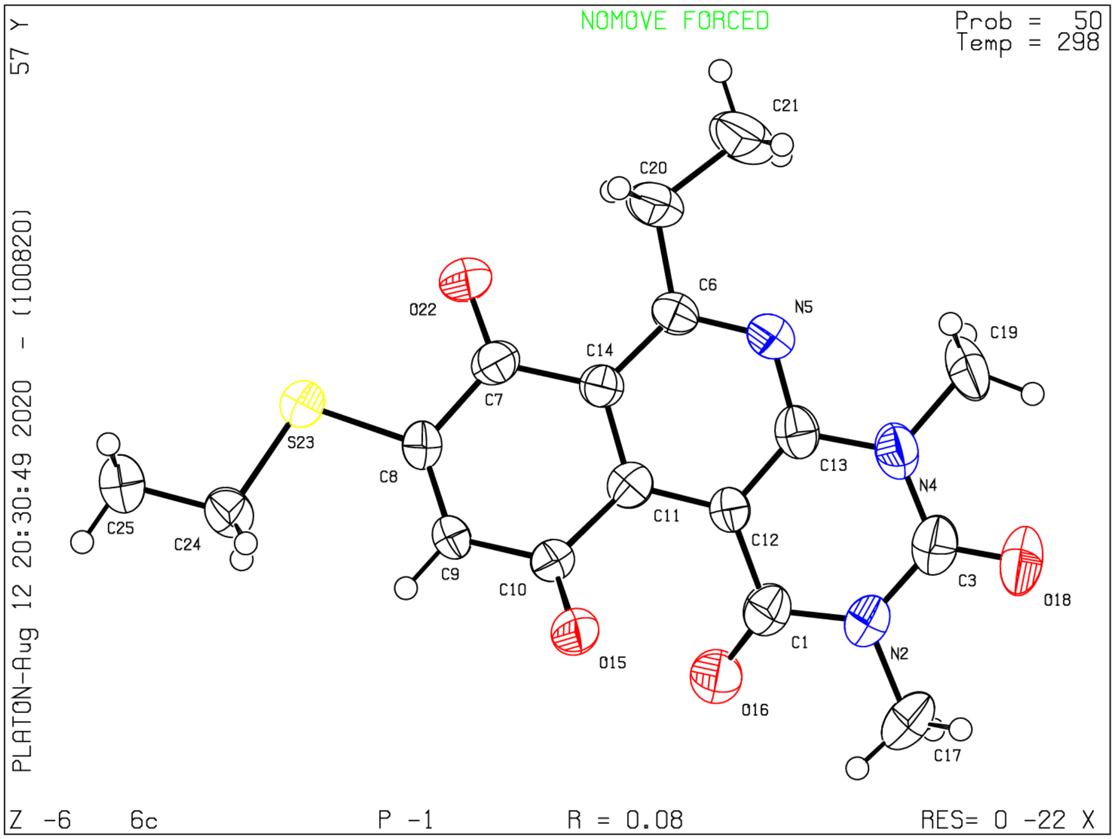

Figure S7 **checkCIF/PLATON report**

You have not supplied any structure factors. As a result the full set of tests cannot be run.

THIS REPORT IS FOR GUIDANCE ONLY. IF USED AS PART OF A REVIEW PROCEDURE FOR PUBLICATION, IT SHOULD NOT REPLACE THE EXPERTISE OF AN EXPERIENCED CRYSTALLOGRAPHIC REFEREE.

No syntax errors found. CIF dictionary Interpreting this report

# Datablock: 7c

```
Bond precision:  C-C = 0.0056 A          Wavelength=1.54178

Cell:            a=7.6795(2)     b=16.6845(4)       c=15.1553(5)
                 alpha=90        beta=95.638(2)     gamma=90
Temperature:     298 K

                Calculated                Reported
Volume          1932.43(9)                1932.43(9)
Space group     P 21/c                    P2(1)/c
Hall group      -P 2ybc                   -P 2ybc
Moiety formula  C21 H17 Cl N4 O4          C21 H17 Cl N4 O4
Sum formula     C21 H17 Cl N4 O4          C21 H17 Cl N4 O4
Mr              424.84                    424.84
Dx,g cm-3       1.460                     1.460
Z               4                         4
Mu (mm-1)       2.080                     2.080
F000            880.0                     880.0
F000'           884.11
h,k,lmax        9,19,18                   9,19,17
Nref            3418                      3405
Tmin,Tmax       0.779,0.812               0.622,0.753
Tmin'           0.779

Correction method= # Reported T Limits: Tmin=0.622 Tmax=0.753
AbsCorr = MULTI-SCAN

Data completeness= 0.996          Theta(max)= 66.758

R(reflections)= 0.0684( 2086)     wR2(reflections)= 0.1789( 3405)

S = 1.062                  Npar= 275
```

The following ALERTS were generated. Each ALERT has the format
**test-name_ALERT_alert-type_alert-level**.
Click on the hyperlinks for more details of the test.

Figure S7

**Alert level C**

```
PLAT340_ALERT_3_C Low Bond Precision on  C-C Bonds ...............    0.00556 Ang.
PLAT410_ALERT_2_C Short Intra H...H Contact  H6A      ..H9       .       1.99 Ang.
                                                  x,y,z  =      1_555 Check
```

**Alert level G**

```
PLAT007_ALERT_5_G Number of Unrefined Donor-H Atoms .............          1 Report
PLAT432_ALERT_2_G Short Inter X...Y Contact  O16      ..C19              2.89 Ang.
                                      2-x,-1/2+y,3/2-z  =      2_746 Check
PLAT883_ALERT_1_G No Info/Value for _atom_sites_solution_primary .     Please Do !
```

```
   0 ALERT level A = Most likely a serious problem - resolve or explain
   0 ALERT level B = A potentially serious problem, consider carefully
   2 ALERT level C = Check. Ensure it is not caused by an omission or oversight
   3 ALERT level G = General information/check it is not something unexpected

   1 ALERT type 1 CIF construction/syntax error, inconsistent or missing data
   2 ALERT type 2 Indicator that the structure model may be wrong or deficient
   1 ALERT type 3 Indicator that the structure quality may be low
   0 ALERT type 4 Improvement, methodology, query or suggestion
   1 ALERT type 5 Informative message, check
```

It is advisable to attempt to resolve as many as possible of the alerts in all categories. Often the minor alerts point to easily fixed oversights, errors and omissions in your CIF or refinement strategy, so attention to these fine details can be worthwhile. In order to resolve some of the more serious problems it may be necessary to carry out additional measurements or structure refinements. However, the purpose of your study may justify the reported deviations and the more serious of these should normally be commented upon in the discussion or experimental section of a paper or in the "special_details" fields of the CIF. checkCIF was carefully designed to identify outliers and unusual parameters, but every test has its limitations and alerts that are not important in a particular case may appear. Conversely, the absence of alerts does not guarantee there are no aspects of the results needing attention. It is up to the individual to critically assess their own results and, if necessary, seek expert advice.

**Publication of your CIF in IUCr journals**

A basic structural check has been run on your CIF. These basic checks will be run on all CIFs submitted for publication in IUCr journals (*Acta Crystallographica*, *Journal of Applied Crystallography*, *Journal of Synchrotron Radiation*); however, if you intend to submit to *Acta Crystallographica Section C* or *E* or *IUCrData*, you should make sure that full publication checks are run on the final version of your CIF prior to submission.

**Publication of your CIF in other journals**

Please refer to the *Notes for Authors* of the relevant journal for any special instructions relating to CIF submission.

**PLATON version of 10/08/2020; check.def file version of 06/08/2020**

Figure S7 **Datablock 7c** - ellipsoid plot

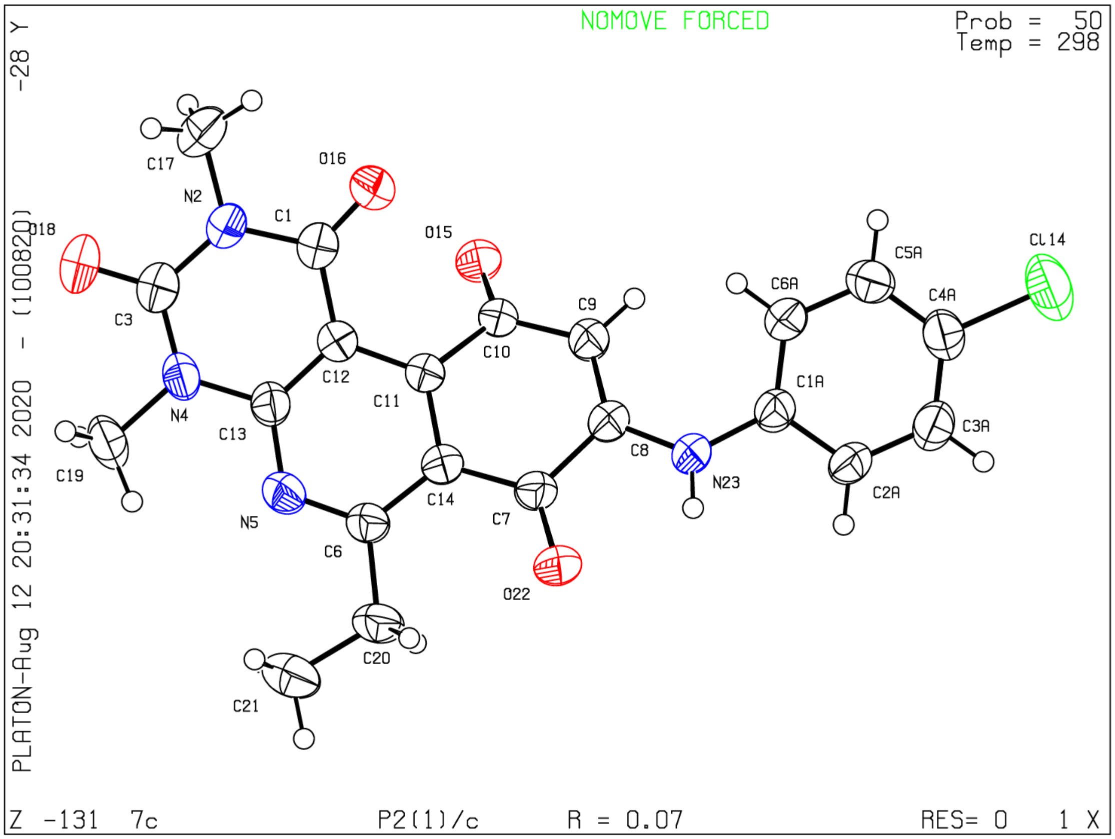

Figure S8 **checkCIF/PLATON report**

You have not supplied any structure factors. As a result the full set of tests cannot be run.

THIS REPORT IS FOR GUIDANCE ONLY. IF USED AS PART OF A REVIEW PROCEDURE FOR PUBLICATION, IT SHOULD NOT REPLACE THE EXPERTISE OF AN EXPERIENCED CRYSTALLOGRAPHIC REFEREE.

No syntax errors found. CIF dictionary Interpreting this report

# Datablock: 8c

---

```
Bond precision:  C-C = 0.0033 A         Wavelength=1.54178

Cell:            a=7.6848(2)    b=16.8295(5)     c=15.2751(5)
                 alpha=90       beta=95.860(2)   gamma=90
Temperature:     298 K
```

| | Calculated | Reported |
|---|---|---|
| Volume | 1965.23(10) | 1965.23(10) |
| Space group | P 21/c | P2(1)/c |
| Hall group | -P 2ybc | -P 2ybc |
| Moiety formula | C21 H17 Br N4 O4 | C21 H17 Br N4 O4 |
| Sum formula | C21 H17 Br N4 O4 | C21 H17 Br N4 O4 |
| Mr | 469.29 | 469.30 |
| Dx,g cm-3 | 1.586 | 1.586 |
| Z | 4 | 4 |
| Mu (mm-1) | 3.177 | 3.177 |
| F000 | 952.0 | 952.0 |
| F000’ | 951.93 | |
| h,k,lmax | 9,20,18 | 9,19,18 |
| Nref | 3464 | 3443 |
| Tmin,Tmax | 0.717,0.728 | 0.537,0.753 |
| Tmin’ | 0.650 | |

```
Correction method= # Reported T Limits: Tmin=0.537 Tmax=0.753
AbsCorr = MULTI-SCAN

Data completeness= 0.994         Theta(max)= 66.641

R(reflections)= 0.0396( 2971)    wR2(reflections)= 0.1059( 3443)

S = 1.074                 Npar= 275
```

---

The following ALERTS were generated. Each ALERT has the format
**test-name_ALERT_alert-type_alert-level**.
Click on the hyperlinks for more details of the test.

Figure S8

**Alert level G**

```
PLAT007_ALERT_5_G Number of Unrefined Donor-H Atoms .............          1 Report
PLAT432_ALERT_2_G Short Inter X...Y Contact  O16      ..C19              2.96 Ang.
                                    2-x,-1/2+y,3/2-z  =      2_746 Check
PLAT434_ALERT_2_G Short Inter HL..HL Contact Br14     ..Br14             3.55 Ang.
                                           -x,-y,1-z  =      3_556 Check
PLAT883_ALERT_1_G No Info/Value for _atom_sites_solution_primary .     Please Do !
```

```
   0 ALERT level A = Most likely a serious problem - resolve or explain
   0 ALERT level B = A potentially serious problem, consider carefully
   0 ALERT level C = Check. Ensure it is not caused by an omission or oversight
   4 ALERT level G = General information/check it is not something unexpected

   1 ALERT type 1 CIF construction/syntax error, inconsistent or missing data
   2 ALERT type 2 Indicator that the structure model may be wrong or deficient
   0 ALERT type 3 Indicator that the structure quality may be low
   0 ALERT type 4 Improvement, methodology, query or suggestion
   1 ALERT type 5 Informative message, check
```

It is advisable to attempt to resolve as many as possible of the alerts in all categories. Often the minor alerts point to easily fixed oversights, errors and omissions in your CIF or refinement strategy, so attention to these fine details can be worthwhile. In order to resolve some of the more serious problems it may be necessary to carry out additional measurements or structure refinements. However, the purpose of your study may justify the reported deviations and the more serious of these should normally be commented upon in the discussion or experimental section of a paper or in the "special_details" fields of the CIF. checkCIF was carefully designed to identify outliers and unusual parameters, but every test has its limitations and alerts that are not important in a particular case may appear. Conversely, the absence of alerts does not guarantee there are no aspects of the results needing attention. It is up to the individual to critically assess their own results and, if necessary, seek expert advice.

**Publication of your CIF in IUCr journals**

A basic structural check has been run on your CIF. These basic checks will be run on all CIFs submitted for publication in IUCr journals (*Acta Crystallographica*, *Journal of Applied Crystallography*, *Journal of Synchrotron Radiation*); however, if you intend to submit to *Acta Crystallographica Section C* or *E* or *IUCrData*, you should make sure that full publication checks are run on the final version of your CIF prior to submission.

**Publication of your CIF in other journals**

Please refer to the *Notes for Authors* of the relevant journal for any special instructions relating to CIF submission.

**PLATON version of 10/08/2020; check.def file version of 06/08/2020**

**Datablock 8c** - ellipsoid plot

Figure S8

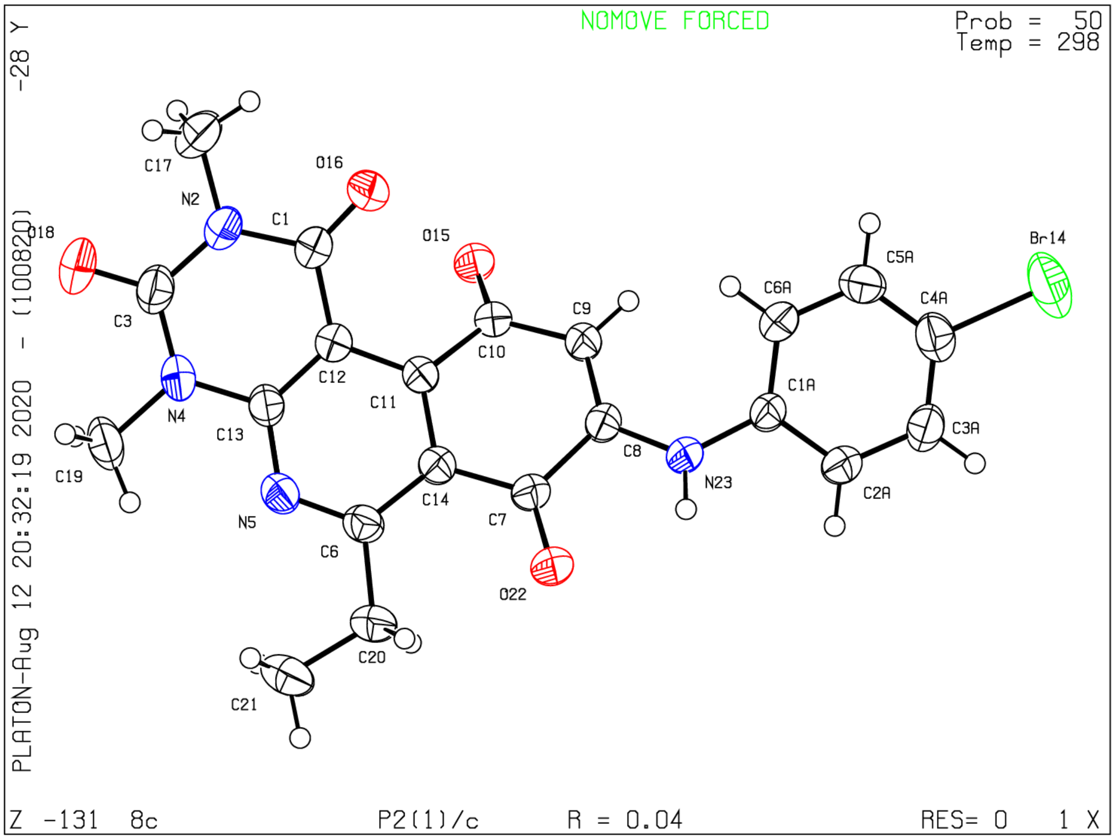

Figure S9 **checkCIF/PLATON report**

You have not supplied any structure factors. As a result the full set of tests cannot be run.

THIS REPORT IS FOR GUIDANCE ONLY. IF USED AS PART OF A REVIEW PROCEDURE FOR PUBLICATION, IT SHOULD NOT REPLACE THE EXPERTISE OF AN EXPERIENCED CRYSTALLOGRAPHIC REFEREE.

No syntax errors found. CIF dictionary Interpreting this report

## Datablock: 11c

```
Bond precision:  C-C = 0.0081 A          Wavelength=1.54178

Cell:             a=24.2195(6)      b=7.5475(2)     c=21.3877(6)
                  alpha=90          beta=90         gamma=90
Temperature:      298 K

                Calculated                 Reported
Volume          3909.60(18)                3909.60(18)
Space group     P b c n                    P b c n
Hall group      -P 2n 2ab                  -P 2n 2ab
Moiety formula  C20 H14 Br N3 O4 S         C20 H14 Br N3 O4 S
Sum formula     C20 H14 Br N3 O4 S         C20 H14 Br N3 O4 S
Mr              472.30                     472.31
Dx,g cm-3       1.605                      1.605
Z               8                          8
Mu (mm-1)       4.156                      4.156
F000            1904.0                     1904.0
F000'           1906.21
h,k,lmax        28,8,25                    28,8,25
Nref            3455                       3427
Tmin,Tmax       0.638,0.660                0.442,0.753
Tmin'           0.578

Correction method= # Reported T Limits: Tmin=0.442 Tmax=0.753
AbsCorr = NONE

Data completeness= 0.992          Theta(max)= 66.747

R(reflections)= 0.0654( 2228)     wR2(reflections)= 0.1621( 3427)

S = 1.090                  Npar= 266
```

The following ALERTS were generated. Each ALERT has the format
**test-name_ALERT_alert-type_alert-level**.
Click on the hyperlinks for more details of the test.

Figure S9

**Alert level C**

```
PLAT341_ALERT_3_C Low Bond Precision on  C-C Bonds ...............    0.00812 Ang.
PLAT601_ALERT_2_C Unit Cell Contains Solvent Accessible VOIDS of .         38 Ang**3
```

**Alert level G**

```
PLAT083_ALERT_2_G SHELXL Second Parameter in WGHT  Unusually Large      10.26 Why ?
PLAT432_ALERT_2_G Short Inter X...Y Contact  O16      ..C3               3.01 Ang.
                                           3/2-x,1/2+y,z  =      8_765 Check
PLAT883_ALERT_1_G No Info/Value for _atom_sites_solution_primary .     Please Do !
```

```
  0 ALERT level A = Most likely a serious problem - resolve or explain
  0 ALERT level B = A potentially serious problem, consider carefully
  2 ALERT level C = Check. Ensure it is not caused by an omission or oversight
  3 ALERT level G = General information/check it is not something unexpected

  1 ALERT type 1 CIF construction/syntax error, inconsistent or missing data
  3 ALERT type 2 Indicator that the structure model may be wrong or deficient
  1 ALERT type 3 Indicator that the structure quality may be low
  0 ALERT type 4 Improvement, methodology, query or suggestion
  0 ALERT type 5 Informative message, check
```

It is advisable to attempt to resolve as many as possible of the alerts in all categories. Often the minor alerts point to easily fixed oversights, errors and omissions in your CIF or refinement strategy, so attention to these fine details can be worthwhile. In order to resolve some of the more serious problems it may be necessary to carry out additional measurements or structure refinements. However, the purpose of your study may justify the reported deviations and the more serious of these should normally be commented upon in the discussion or experimental section of a paper or in the "special_details" fields of the CIF. checkCIF was carefully designed to identify outliers and unusual parameters, but every test has its limitations and alerts that are not important in a particular case may appear. Conversely, the absence of alerts does not guarantee there are no aspects of the results needing attention. It is up to the individual to critically assess their own results and, if necessary, seek expert advice.

**Publication of your CIF in IUCr journals**

A basic structural check has been run on your CIF. These basic checks will be run on all CIFs submitted for publication in IUCr journals (*Acta Crystallographica*, *Journal of Applied Crystallography*, *Journal of Synchrotron Radiation*); however, if you intend to submit to *Acta Crystallographica Section C* or *E* or *IUCrData*, you should make sure that full publication checks are run on the final version of your CIF prior to submission.

**Publication of your CIF in other journals**

Please refer to the *Notes for Authors* of the relevant journal for any special instructions relating to CIF submission.

**PLATON version of 10/08/2020; check.def file version of 06/08/2020**

Figure S9 **Datablock 11c** - ellipsoid plot

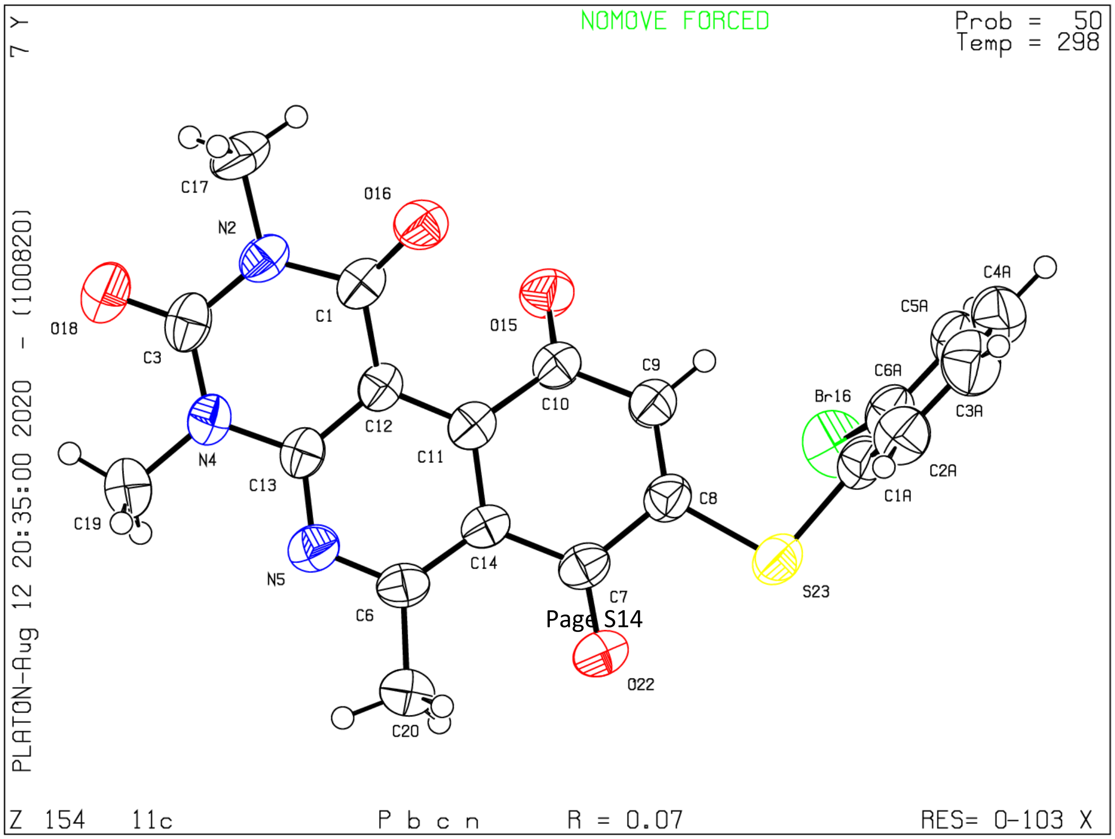


Page S14

Figure S10 **checkCIF/PLATON report**

You have not supplied any structure factors. As a result the full set of tests cannot be run.

THIS REPORT IS FOR GUIDANCE ONLY. IF USED AS PART OF A REVIEW PROCEDURE FOR PUBLICATION, IT SHOULD NOT REPLACE THE EXPERTISE OF AN EXPERIENCED CRYSTALLOGRAPHIC REFEREE.

No syntax errors found. CIF dictionary Interpreting this report

# Datablock: 19c

---

```
Bond precision:  C-C = 0.0032 A          Wavelength=1.54178

Cell:            a=4.5196(2)    b=26.8428(13)     c=15.3585(10)
                 alpha=90       beta=95.006(4)    gamma=90
Temperature:     298 K

                Calculated                Reported
Volume          1856.17(17)               1856.17(17)
Space group     P 21/n                    P2(1)/n
Hall group      -P 2yn                    -P 2yn
Moiety formula  C19 H21 N3 O4 S           C19 H21 N3 O4 S
Sum formula     C19 H21 N3 O4 S           C19 H21 N3 O4 S
Mr              387.45                    387.45
Dx,g cm-3       1.387                     1.386
Z               4                         4
Mu (mm-1)       1.816                     1.816
F000            816.0                     816.0
F000’           819.74
h,k,lmax        5,32,18                   5,31,18
Nref            3318                      3269
Tmin,Tmax       0.819,0.849               0.529,0.753
Tmin’           0.819

Correction method= # Reported T Limits: Tmin=0.529 Tmax=0.753
AbsCorr = MULTI-SCAN

Data completeness= 0.985          Theta(max)= 66.839

R(reflections)= 0.0441( 2724)     wR2(reflections)= 0.1155( 3269)

S = 1.046                   Npar= 249
```

---

The following ALERTS were generated. Each ALERT has the format
**test-name_ALERT_alert-type_alert-level**.
Click on the hyperlinks for more details of the test.

Figure S10 **Alert level G**

```
PLAT335_ALERT_2_G Check Large C6 Ring C-C Range C7       -C14           0.17 Ang.
PLAT883_ALERT_1_G No Info/Value for _atom_sites_solution_primary .     Please Do !
```

```
  0 ALERT level A = Most likely a serious problem - resolve or explain
  0 ALERT level B = A potentially serious problem, consider carefully
  0 ALERT level C = Check. Ensure it is not caused by an omission or oversight
  2 ALERT level G = General information/check it is not something unexpected

  1 ALERT type 1 CIF construction/syntax error, inconsistent or missing data
  1 ALERT type 2 Indicator that the structure model may be wrong or deficient
  0 ALERT type 3 Indicator that the structure quality may be low
  0 ALERT type 4 Improvement, methodology, query or suggestion
  0 ALERT type 5 Informative message, check
```

It is advisable to attempt to resolve as many as possible of the alerts in all categories. Often the minor alerts point to easily fixed oversights, errors and omissions in your CIF or refinement strategy, so attention to these fine details can be worthwhile. In order to resolve some of the more serious problems it may be necessary to carry out additional measurements or structure refinements. However, the purpose of your study may justify the reported deviations and the more serious of these should normally be commented upon in the discussion or experimental section of a paper or in the "special_details" fields of the CIF. checkCIF was carefully designed to identify outliers and unusual parameters, but every test has its limitations and alerts that are not important in a particular case may appear. Conversely, the absence of alerts does not guarantee there are no aspects of the results needing attention. It is up to the individual to critically assess their own results and, if necessary, seek expert advice.

**Publication of your CIF in IUCr journals**

A basic structural check has been run on your CIF. These basic checks will be run on all CIFs submitted for publication in IUCr journals (*Acta Crystallographica*, *Journal of Applied Crystallography*, *Journal of Synchrotron Radiation*); however, if you intend to submit to *Acta Crystallographica Section C* or *E* or *IUCrData*, you should make sure that full publication checks are run on the final version of your CIF prior to submission.

**Publication of your CIF in other journals**

Please refer to the *Notes for Authors* of the relevant journal for any special instructions relating to CIF submission.

**PLATON version of 10/08/2020; check.def file version of 06/08/2020**

Figure S10 **Datablock 19c** - ellipsoid plot

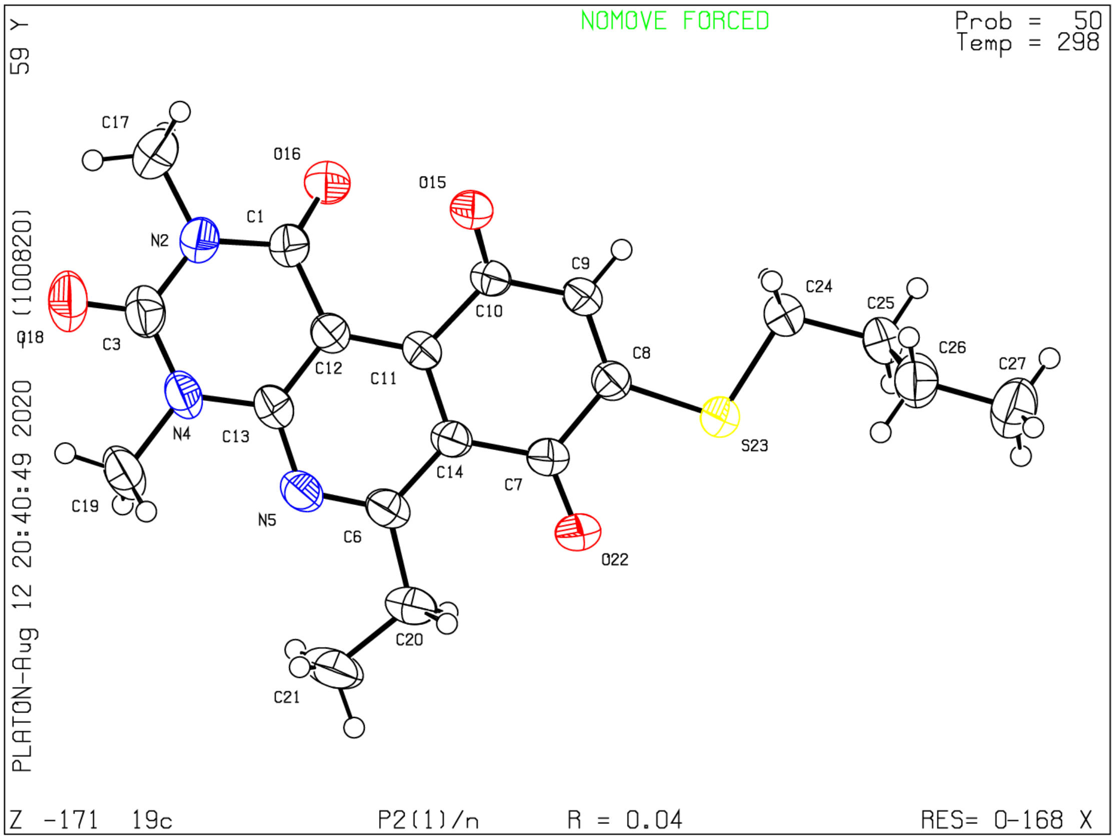

Figure S11 **checkCIF/PLATON report**

You have not supplied any structure factors. As a result the full set of tests cannot be run.

THIS REPORT IS FOR GUIDANCE ONLY. IF USED AS PART OF A REVIEW PROCEDURE FOR PUBLICATION, IT SHOULD NOT REPLACE THE EXPERTISE OF AN EXPERIENCED CRYSTALLOGRAPHIC REFEREE.

No syntax errors found. CIF dictionary Interpreting this report

# Datablock: 20c

```
Bond precision:  C-C = 0.0021 A         Wavelength=1.54178

Cell:            a=8.7845(3)    b=10.4767(4)      c=21.3483(10)
                 alpha=90       beta=99.351(3)    gamma=90
Temperature:     298 K

                Calculated                Reported
Volume          1938.63(14)               1938.63(14)
Space group     P 21/c                    P2(1)/c
Hall group      -P 2ybc                   -P 2ybc
Moiety formula  C21 H19 N5 O4             C21 H19 N5 O4
Sum formula     C21 H19 N5 O4             C21 H19 N5 O4
Mr              405.41                    405.41
Dx,g cm-3       1.389                     1.389
Z               4                         4
Mu (mm-1)       0.821                     0.821
F000            848.0                     848.0
F000'           850.74
h,k,lmax        10,12,25                  10,12,25
Nref            3453                      3426
Tmin,Tmax       0.906,0.914               0.488,0.753
Tmin'           0.906

Correction method= # Reported T Limits: Tmin=0.488 Tmax=0.753
AbsCorr = MULTI-SCAN

Data completeness= 0.992          Theta(max)= 66.788

R(reflections)= 0.0481( 3178)     wR2(reflections)= 0.1334( 3426)

S = 1.043                   Npar= 276
```

The following ALERTS were generated. Each ALERT has the format
**test-name_ALERT_alert-type_alert-level**.
Click on the hyperlinks for more details of the test.

Figure S11 **Alert level C**

```
PLAT220_ALERT_2_C NonSolvent   Resd 1  C   Ueq(max)/Ueq(min) Range        3.1 Ratio
PLAT242_ALERT_2_C Low    'MainMol' Ueq as Compared to Neighbors of        C20 Check
```

**Alert level G**

```
PLAT007_ALERT_5_G Number of Unrefined Donor-H Atoms .............          3 Report
PLAT432_ALERT_2_G Short Inter X...Y Contact  O15      ..C7             2.92 Ang.
                                          1-x,1-y,1-z  =      3_666 Check
PLAT432_ALERT_2_G Short Inter X...Y Contact  O22      ..C3             2.98 Ang.
                                           -x,1-y,1-z  =      3_566 Check
PLAT883_ALERT_1_G No Info/Value for _atom_sites_solution_primary .     Please Do !
PLAT933_ALERT_2_G Number of OMIT Records in Embedded .res File ...          1 Note
```

0 **ALERT level A** = Most likely a serious problem - resolve or explain
0 **ALERT level B** = A potentially serious problem, consider carefully
2 **ALERT level C** = Check. Ensure it is not caused by an omission or oversight
5 **ALERT level G** = General information/check it is not something unexpected

1 ALERT type 1 CIF construction/syntax error, inconsistent or missing data
5 ALERT type 2 Indicator that the structure model may be wrong or deficient
0 ALERT type 3 Indicator that the structure quality may be low
0 ALERT type 4 Improvement, methodology, query or suggestion
1 ALERT type 5 Informative message, check

---

It is advisable to attempt to resolve as many as possible of the alerts in all categories. Often the minor alerts point to easily fixed oversights, errors and omissions in your CIF or refinement strategy, so attention to these fine details can be worthwhile. In order to resolve some of the more serious problems it may be necessary to carry out additional measurements or structure refinements. However, the purpose of your study may justify the reported deviations and the more serious of these should normally be commented upon in the discussion or experimental section of a paper or in the "special_details" fields of the CIF. checkCIF was carefully designed to identify outliers and unusual parameters, but every test has its limitations and alerts that are not important in a particular case may appear. Conversely, the absence of alerts does not guarantee there are no aspects of the results needing attention. It is up to the individual to critically assess their own results and, if necessary, seek expert advice.

**Publication of your CIF in IUCr journals**

A basic structural check has been run on your CIF. These basic checks will be run on all CIFs submitted for publication in IUCr journals (*Acta Crystallographica*, *Journal of Applied Crystallography*, *Journal of Synchrotron Radiation*); however, if you intend to submit to *Acta Crystallographica Section C* or *E* or *IUCrData*, you should make sure that full publication checks are run on the final version of your CIF prior to submission.

**Publication of your CIF in other journals**

Please refer to the *Notes for Authors* of the relevant journal for any special instructions relating to CIF submission.

Figure S11

**PLATON version of 10/08/2020; check.def file version of 06/08/2020**

**Datablock 20c** - ellipsoid plot

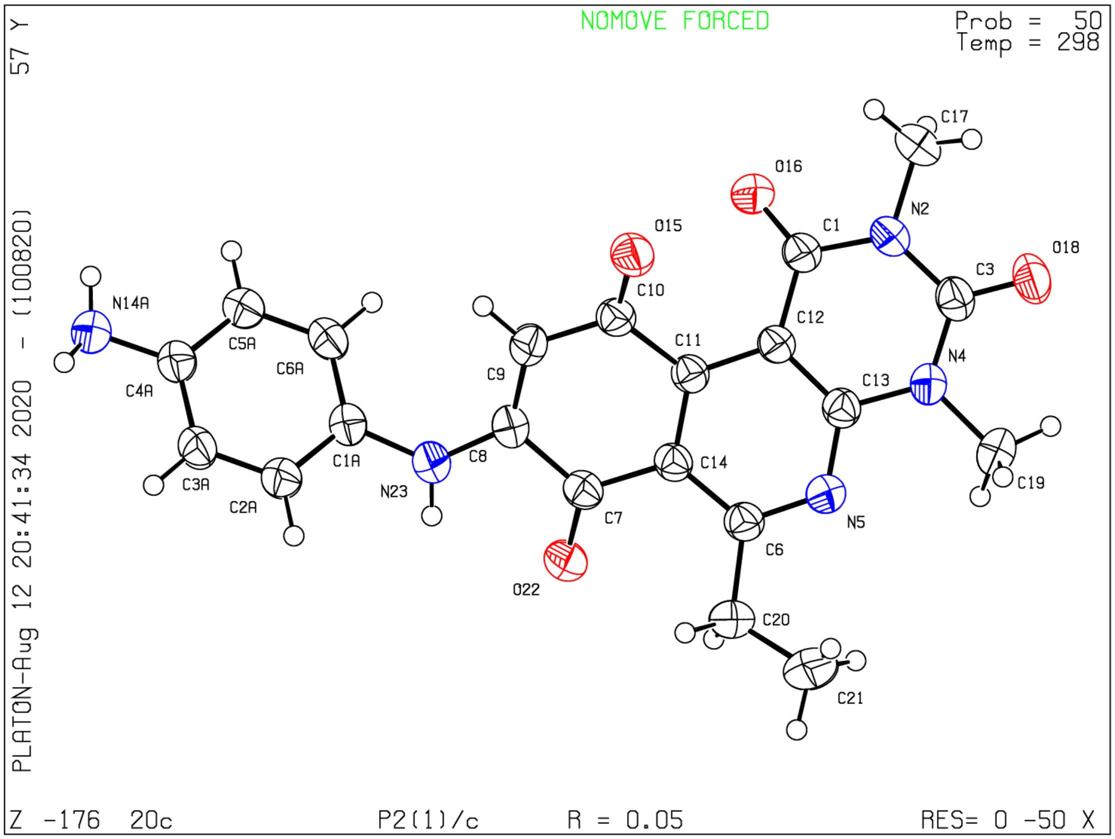

Supplement: Supplementary file 1 [file antibiotics-12-01065-s001.zip › antibiotics-2456008-supplementary.pdf]
